# Outcomes Associated With Airway Management of Adult Trauma Patients Admitted to Surgical Intensive Care

**DOI:** 10.7759/cureus.75875

**Published:** 2024-12-17

**Authors:** Elizabeth Cunningham, Danielle O'Rourke, Karen Fitzgerald, Nader Azab, Lauren Rothburd, Brian Awgul, Christopher Raio, Lauren R Klein, Catherine Caronia, Heather Reens, Theresa Drucker, Fathia Qandeel, Amirun Mahia, Anupreet Kaur, Sarah Eckardt, Patricia A Eckardt

**Affiliations:** 1 Surgical Intensive Care, Good Samaritan University Hospital, West Islip, USA; 2 Performance Improvement, Good Samaritan University Hospital, West Islip, USA; 3 Quality Improvement, Good Samaritan University Hospital, West Islip, USA; 4 Intensive Care, Good Samaritan University Hospital, West Islip, USA; 5 Trauma, Good Samaritan University Hospital, West Islip, USA; 6 Medical Library, Good Samaritan University Hospital, West Islip, USA; 7 Emergency Medicine, Good Samaritan University Hospital, West Islip, USA; 8 Pediatrics, Good Samaritan University Hospital, West Islip, USA; 9 Nursing, Molloy University, Rockville Centre, USA; 10 Research, Good Samaritan University Hospital, West Islip, USA; 11 Medicine, City University of New York, New York City, USA; 12 Data Science, Eckardt & Eckardt Consulting, St. James, USA; 13 Nursing, Good Samaritan University Hospital, West Islip, USA

**Keywords:** advanced airway management, airway management, critical care, early intubation, late intubation, mechanical ventilation, social determinants of health (sdoh), surgical intensive care unit (sicu), tracheostomy, endotracheal intubation

## Abstract

Introduction: Advanced airway management and ventilation of trauma patients are often needed during acute stabilization and resuscitation and later, in those admitted. In addition to endotracheal intubation for advanced airway management, tracheostomy is commonly used in critically ill patients when prolonged mechanical ventilation is required. However, the outcomes associated with airway management approaches and the timing of a tracheostomy in critically ill patients are mixed. This protocol intended to compare the effect of tracheostomy in major trauma patients vs. management with non-invasive techniques and endotracheal intubation during admission, examine complications and outcomes associated with the three types of airway management approaches, and explore the association of clinical and social determinants of health variables with complications in patients requiring advanced airway management.

Methods: A total of 911 adult trauma patients admitted to a Level 1 trauma center surgical intensive care unit (SICU) were included in this retrospective, single-center, quantitative study from 2019 to 2021. Descriptive and correlational analyses were used to examine outcomes of ventilator days, length of stay, pneumonia, readmission, mortality, and associations with the airway management approach. The outcomes of ventilator days and length of stay were compared between groups with a one-way ANOVA, and differences between groups on outcomes of pneumonia, readmission, and mortality were estimated using crosstabulations and chi-square (x²) statistics. Hypothesized relationships of clinical and social determinants of health variables associated with outcomes of ventilator days, hospital length of stay, pneumonia, readmission, and mortality in patients requiring advanced airway management ≥ four days were estimated.

Results: There was no significant difference in outcomes of pneumonia and mortality between the advanced airway management groups (p=0.856 and p=0.167, respectively). There were significant differences in ventilator days, length of stay (LOS), and readmission. Between the groups: endotracheal intubation only, early (<10 days post-intubation) tracheostomy, and late (>10 days post-intubation) tracheostomy in SICU patients (p <0.001, p=0.028, and p=0.003, respectively). Specifically, patients in the early tracheostomy group had a higher readmission rate (33.3%) as compared to endotracheal tube patients (2.3%) and late tracheostomy patients (0.0%). Social determinants of health variables (smoking and functional dependence) were also significantly correlated with readmission in the early tracheostomy and endotracheal tube airway management groups (p=.047 and p=.022, respectively). Additionally, clinical variables of injury severity scores, ED arrival systolic blood pressure (SBP), and presence of pre-existing comorbidities were found to be significantly associated with complications of pneumonia, readmission, and mortality within the patients (n=229) requiring advanced airway approaches.

Conclusion: Adult trauma patients with early tracheostomy airway management may experience a higher readmission rate related to the complexity of their injuries than patients managed with endotracheal intubation or late tracheostomy. Clinical and social determinants of health factors may be associated with complications. Further studies examining these associations in larger samples are needed to examine the validity of these findings.

## Introduction

Advanced airway management and ventilation of major trauma patients is often needed during acute stabilization and resuscitation and later in those admitted to the surgical intensive care unit (SICU) [1]. Tracheostomy is commonly used in critically ill patients, including major trauma patients when prolonged mechanical ventilation is required due to acute respiratory failure or airway problems. However, the complications and outcomes associated with the type of advanced airway management and timing of a tracheostomy (early tracheostomy defined as < 10 days post intubation, and late tracheostomy defined as ≥ 10 days post-intubation) in critically ill patients, such as ventilator days, length of stay (LOS), pneumonia, readmission, and mortality, are mixed [2-4]. Specifically, the impact of airway management on complications and outcomes in trauma patients varies across studies. One systematic review and meta-analysis (n=20 studies) found in a sample size of 4,446 patients that mortality was not lower in trauma patients with early tracheostomy compared to those with late tracheostomy or endotracheal tube only. However, the same study found early tracheostomy to be associated with a significantly reduced hospital LOS, shorter ventilator days, and a lower risk of pneumonia [5]. Another meta-analysis of nine randomized clinical trials with 2,072 participants found that in patients requiring prolonged mechanical ventilation, the early tracheostomy group showed no significant difference in clinical outcomes compared to that of the late tracheostomy or intubation-only groups. Specifically, short-term and long-term mortality were not reduced (p = 0.14 and p = 0.27, respectively). Additionally, early tracheostomy was not associated with the incidence of pneumonia (p=0.27) or duration of mechanical ventilation (p=0.19) [6]. Of note, the incidence of complications associated with advanced airway management may vary by mechanism of injury and other associated interventions. For patients with severe traumatic brain injury or stroke, one five-year retrospective cohort study (n=135) found earlier tracheostomy was associated with a decrease in mechanical ventilation days [7]. Similarly, a multicenter retrospective review of 124 patients with blunt chest trauma as the mechanism of injury found early tracheostomy was correlated with a statistically significant reduction in the duration of mechanical ventilation [8]. However, in multiple retrospective studies, patients undergoing early tracheostomy have been younger with either higher injury severity scores, fewer comorbidities, or identified deteriorating clinical factors as compared to patients with late tracheostomy and endotracheal intubation airway management groups [2,9,10]. The only randomized control trial to date for early tracheostomy, defined as tracheostomy within three days on a ventilator, consisted of patients who had experienced a stroke (n=60) and found no differences in primary outcome intensive care unit (ICU) LOS. However, ICU mortality and six-month mortality were significantly lower in the early tracheostomy group with ICU deaths (three (10%) versus 14 (47%); p < 0.01) and six-month mortality deaths (eight (27%) versus 18 (60%); p = 0.02, respectively). The lack of consistency in findings impacts the ability of clinicians to identify patients at risk for complications and to take action to prevent them [11-13]. Additionally, recent studies have found that social determinants of health (SDoH), in addition to clinical factors, have been associated with outcomes within critically ill populations [14,15]. Specifically in adult trauma patients, smoking and functional dependence have been associated with increased mortality [16,17]. Accordingly, our study attempts to fill this gap of evidence associated with the conflicting nature of past research findings by focusing on trauma patients requiring advanced airway management approaches ≥ four days of duration.

Therefore, the objectives of this study were as follows: to compare the outcomes of tracheostomy and endotracheal intubation for advanced airway management in adult major trauma patients admitted to the SICU; to examine the complications (e.g., pneumonia, readmission, mortality) and length of hospital stay associated with different airway management strategies; and to explore the relationship between clinical factors, such as trauma mechanism of injury (MOI), arrival Glasgow Coma Scale (GCS) score, Injury Severity Score (ISS), vital signs, and SDoH, including current smoker and functional dependence in patients requiring advanced airway management.

## Materials and methods

Study design

This retrospective, single-center, quantitative study was conducted by the Department of Trauma Surgery at Good Samaritan University Hospital, a Level 1 trauma center in Long Island, New York. Of note, in accordance with the US Federal Emergency Medical Treatment & Labor Act (EMTALA) guidelines specific to New York State, all patients arriving via ambulance must be brought to the nearest emergency room, regardless of the institution’s trauma center level, for a medical screening examination. Although the institution utilized for the study was a Level 1 trauma center, it must be understood that patients were brought to the institution because of their proximity to the location, as opposed to requiring services that only a Level 1 trauma center could provide, such as treatment of high-impact injuries and polytrauma patients.

Patients were included in the study if they were aged 17 years or greater with an International Classification of Diseases-10 (ICD-10) code [18] for trauma admitted to the trauma center during the time period from January 1, 2019, through December 31, 2021. Exclusion criteria were patients younger than 17 years of age and patients admitted for a trauma diagnosis to another unit in the hospital or discharged to home during the sampling time frame.

Data collection

After approval from the institutional review board for research with human subjects (approval number: IRB # 22-010), data from patients who met the inclusion criteria were exported from the electronic health record and stored as .csv files in Research Electronic Data Capture (REDCap; Vanderbilt University, Nashville, TN) for primary research record source. Data was then exported into Microsoft Excel (Microsoft Corp., Redmond, WA) for data recoding and transformation. The Office of Management and Budget (OMB) and the National Institutes of Health (NIH) reporting guidelines for race and ethnicity were used in the collection of the demographics in this study as this study is a retrospective study using data collected from participants from the years 2019-2021.

Outcomes and clinical variables

The primary outcome was ventilator days. Secondary outcomes included LOS, pneumonia, readmission, and mortality. The total number of patients included in the study was 911, and this cohort was divided into four groups: those who had airway management by invasive techniques (tracheostomy and endotracheal intubation) and those who did not. The airway management by invasive techniques group was then further subdivided into three groups: the first group consisted of patients whose airway management was by endotracheal intubation only for ≥ four days duration (non-tracheostomy group); the second group consisted of patients admitted to SICU who had a tracheostomy < 10 days after endotracheal intubation (early tracheostomy); and the third group were patients who had a tracheostomy 10 days and > after endotracheal intubation (late tracheostomy). To restrict the advanced airway management groups to patients who would be considered for tracheostomy after mechanical ventilation, the non-tracheostomy group included patients with endotracheal intubation ≥ four days duration [19].

Descriptive and inferential statistics were used to explore and compare the characteristics of patients within each airway management group and then to compare the patients within the advanced airway management groups only. Inferential statistics of one-way analysis of variance (ANOVA) were used for continuous outcome estimates of ventilator days and hospital LOS. Normality within each treatment group was assessed using Shapiro-Wilk tests. The homogeneity of variance across treatment groups was tested using Levene’s statistic. If normality or homogeneity of variance assumptions were violated, a Box-Cox transformation or Welch test was conducted. Cross tabulations were estimated for discrete outcome measures of mortality, readmission, and pneumonia occurrence to examine outcomes of ventilator days, LOS, pneumonia, readmission, and mortality. A subanalysis using point biserial and tetrachoric correlations for continuous and dichotomous predictors, respectively, estimated hypothesized associations of advanced airway management complications (pneumonia, readmission, and mortality) with clinical and SDoH variables within the advanced airway management sample only.

The clinical and demographic risk factors investigated in this study were chosen because they had previously been associated with the risk of increased ventilator days, LOS, pneumonia, readmission, and mortality for adult trauma patients admitted to SICU as described in the background above. Demographic variables collected from electronic medical record (EMR) data included sex, age, and body mass index (BMI). Clinical characteristics included time from injury to ED presentation, trauma activation level, mechanism of injury (MOI), ED disposition, admitting service, ED arrival Glasgow Coma Scale (GCS) score, Injury Severity Score (ISS), vital signs, and common admission laboratory values such as ED hematocrit, ED lactic acid, ED platelet count, and ED blood alcohol level. The SDoH variables were chosen due to their association with complications in adult trauma patient populations.

Statistical analysis

IBM SPSS Statistics for Windows, Version 28 (IBM Corp., Armonk, NY), and Stata Statistical Software: Release 17 (StataCorp LLC, College Station, TX) were used to perform descriptive statistics of central tendency and dispersion and inferential analyses. Inferential analyses include one-way ANOVAs to compare the means and variance between groups, and Chi-square (χ2) and Fisher’s exact tests were used to compare proportional differences between the groups on the nominal variables. Outcome variables between group comparisons included ventilator days, LOS, pneumonia, readmission, and mortality based on differences in airway management. Inferential analyses used a two-tailed approach, and 0.05 was chosen as the a priori critical alpha value. As the missing data mechanism could not be assumed to be missing completely at random (MCAR) or missing at random (MAR), missing data were handled with complete case analysis (CCA).

## Results

Among the 911 patients included in this cohort, the majority were male, White, and non-Hispanic participants (75.90%, 67.60%, and 61.40%, respectively). Additionally, the majority did not have a trauma pre-activation (82.20%), and of the trauma activations, 45.10% were trauma consults, 29.80% were trauma alerts, and 20.70% were code traumas (Table 1).

**Table 1:** Demographic and clinical characteristics of the study sample (n= 911) ^a^Mean, SD; ^b^Frequency; percentage; ^c^The Office of Management and Budget (OMB) and the National Institutes of Health (NIH) reporting guidelines for race and ethnicity were used in the collection of the demographics in this study as this study is a retrospective study using data collected from participants from the years 2019-2021; *p values significant at 0.05 or <; **p values significant at 0.01 or <; ***p values significant at 0.01 or <; Pre-existing comorbidities included advanced directive limiting care, alcohol use disorder, anticoagulant therapy, bleeding disorder, congenital anomalies, current smoker, functionally dependent health status, hypertension, mental/personality disorders, peripheral arterial disease (PAD), pregnancy, and substance use disorder. SD: standard deviation; ED: emergency department; BMI: body mass index; ISS: Injury Severity Score; GCS: Glasgow Coma Score; SBP: systolic blood pressure; DBP: diastolic blood pressure; HR: heart rate; HCT: hematocrit; INR: international normalized ratio; BAL: blood alcohol level; O2: oxygen saturation; PRBC: packed red blood cells; MTP: massive transfusion protocol

Characteristics	Sample
Sex^b^	
Male	691 (75.90%)
Female	220 (24.10%)
Age^a^	59.71 (SD=25.2)
Race^b^	
Asian	13 (2.00%)
Black	79 (12.00%)
White	444 (67.60%)
Other	121 (18.40%)
Ethnicity^b^	
Hispanic	352 (38.60%)
Non-Hispanic^c^	559 (61.40%)
Extrication^b^	
Yes	32 (3.60%)
No	868 (96.40%)
Prehospital arrest^b^	
Yes	16 (8.10%)
No	894 (98.120%)
Trauma team preactivation^b^	
Yes	161 (7.80%)
No	746 (82.20%)
Trauma activation level^b^	
Not activated	41 (4.50%)
Trauma consult	410 (45.10%)
Trauma alert	271 (29.80%)
Code trauma	188 (20.70%)
Trauma type^b^	
Blunt	876 (96.30%)
Penetrating	32 (3.50%)
Burn	2 (0.02%)
Injury region: face, head, neck^b^	
Yes	487 (46.50%)
No	424 (53.50%)
Transfer in^b^	
Yes	125 (13.70%)
No	785 (86.3%)
Time from Injury to ED^b^	
Less than 30 minutes	74 (8.10%)
31-60 minutes	411 (45.10%)
61-90 minutes	67 (7.4%)
91 minutes-12 hours	154 (16.90%)
Greater than (>) 12 hours	175 (19.20%)
Pre-existing comorbidities^b^	
Yes	215 (23.60%)
No	696 (76.40%)
ED BMI^a^	26.68 (SD=19.49)
ED BMI over (>) 40^b^	
Yes	16 (1.50%)
No	811 (75.60%)
ED ISS^a^	13.05 (SD=8.86)
ED ISS Above 15^b^	
Yes	292 (32.30%)
No	613 (67.70%)
ED serum lactate^a^	4.08 (SD=3.93)
ED platelet count^a^	226.59 (SD=86.70)
ED arrival GCS^a^	13.55 (SD=3.14)
ED SBP^a^	136.53 (SD=28.50)
ED DBP^a^	79.34 (SD=21.40)
ED HR^a^	90.04 (SD=23.32)
ED temperature (Fahrenheit)^a^	97.89 (SD=0.99)
ED HCT^a^	38.97 (SD=6.72)
ED INR^a^	1.25 (SD=1.21)
ED blood alcohol level (BAL)^a^	0.11 (SD=0.13)
ED O_2_^a^	96.52 (SD=5.11)
ED supplemental oxygen administered (O_2_)^b^	
Yes	148 (16.7%)
No	739 (83.3%)
ED probability of survival^a^	0.93 (SD=0.14)
Units of PRBC received in less than 24 hr^a^	0.67 (SD=2.41)
Units of plasma received in less than 24 hr^a^	0.26 (SD=1.44)
Units of platelets received in less than 24 hr^a^	0.28 (SD=0.78)
Units of cryoprecipitate received in less than 24 hr^a^	0.07 (SD=0.54)
Administration of VIIA V in less than 24 hr^a^	0.00 (0.00)
Cellsaver less than 24 hr^a^	0.00 (0.00)
MTP^b^	
Yes	21 (2.3%)
No	890 (97.7%)

When stratified by airway management approach (Table 2), 682 (74.90%) patients did not require invasive airway management, and 218 (23.90%) were managed with intubation only. Of the patients who underwent a tracheostomy, three (0.03%) were early (defined as < 10 days after intubation), and eight (0.09%) were late (≥ 10 days after intubation). The results of the univariate analysis demonstrated some differences between those patients requiring advanced airway management and those who did not. Patients with advanced airway management had significantly higher percentages of trauma alert pre-activations as compared to non-advanced airway management patients: (non-invasive airway, 12.40% (n=84); endotracheal intubation, 67.90% (n=148); early tracheostomy, 100.00% (n=3); and late tracheostomy, 50.00% (n=4); p < .001), lower ED arrival Glasgow Coma Scores (non-invasive airway, 14.48 (SD=1.52); endotracheal intubation, 10.83 (SD=4.64); early tracheostomy, 8.33 (SD=6.11); and late tracheostomy, 6.30 (SD=2.38); p<.001), higher ED ISS values (non-invasive airway, 11.52 (SD=6.55); endotracheal intubation, 17.24 (SD=12.42); early tracheostomy, 28.33 (SD=13.65); and late tracheostomy, 24.75 (SD=13.86); p<.001). When the sample was then compared within the subgroups of advanced airway: endotracheal intubation only, early tracheostomy, and late tracheostomy, there were no significant differences noted between the groups on demographics or clinical factors on ED presentation (Table 3).

**Table 2:** Demographic and clinical characteristics of participants listed as per airway management Early tracheostomy = tracheostomy less than 10 days after endotracheal intubation; late tracheostomy = tracheostomy 10 days and greater after endotracheal intubation; ^a^Mean, SD; ^b^Frequency, percentage; ^c^One-way analysis of variance estimates (ANOVA) (f statistic) were conducted for continuous variable; ^d^Crosstabulation chi-square estimates were conducted for categorical data (except when expected count < 5 in a cell Fisher’s exact statistic was estimated); ^e^Inferential statistic not calculated; the chosen critical alpha level for all significance tests was 0.05 or less; *p values significant at 0.05 or <; **p values significant at 0.01 or <; ***p values significant at 0.01 or < SD: standard deviation; ED: emergency department; BMI: body mass index; ISS: Injury Severity Score; GCS: Glasgow Coma Score; SBP: systolic blood pressure; DBP: diastolic blood pressure; HR: heart rate; HCT: hematocrit; INR: international normalized ratio; BAL: blood alcohol level; O2: oxygen saturation; PRBC: packed red blood cells; MTP: massive transfusion protocol

Characteristics	Non-invasive airway management (n=682)	Endotracheal intubation only (n=218)	Early tracheostomy (n=3)	Late tracheostomy (n=8)	p-value^c,d,e^
Sex^b^					.006**^***^
Male	498 (73.00%)	183 (83.90%)	3 (100.00%)	7 (87.50%)	
Female	184 (27.00%)	35 (16.10%)	0 (0.00%)	1 (12.50%)	
Age^a^	61.52 (SD=25.50)	54.31 (SD=23.31)	39.00 (SD=2.83)	48.67 (SD=15.92)	.007**^c^
Race^b^					.238^d^
Asian	8 (1.60%)	5 (3.40%)	0 (0.00%)	0 (0.00%)	
Black	55 (11.00%)	23 (15.50%)	0 (0.00%)	1 (12.5%)	
White	348 (69.50%)	92 (62.20%)	2 (66.70%)	5 (62.5%)	
Other	90 (18.00%)	28 (18.90%)	1 (33.30%)	2 (25.00%)	
Ethnicity^b^					.200^d^
Hispanic	251 (36.80%)	96 (44.0%)	2 (66.70%)	3 (37.50%)	
Non-Hispanic	431 (63.20%)	122 (56.0%)	1 (33.30%)	5 (62.50%)	
Extrication^b^					.018*^d^
Yes	20 (2.90%)	10 (4.60%)	0 (0.00%)	2 (25.00%)	
No	662 (97.10%)	208 (95.40%)	3 (100.00%)	6 (75.00%)	
Prehospital arrest^b^					<.001***^d^
Yes	5 (0.70%)	10 (4.60%)	1 (33.30%)	0 (0.00%)	
No	677 (99.30%)	208 (95.40%)	2 (66.70%)	8 (100.00%)	
Trauma team preactivation^b^					<.001***^d^
Yes	84 (12.40%)	148 (67.90%)	3 (100.00%)	4 (50.00%)	
No	594 (87.60%)	70 (32.10%)	0 (0.00%)	4 (50.00%)	
Trauma activation level^b^					<.001***^d^
Not activated	28 (4.00%)	14 (6.40%)	0 (0.00%)	0 (0.00%)	
Trauma consult	355 (52.10%)	53 (24.30%)	0 (0.00%)	2 (25.00%)	
Trauma alert	221 (32.50%)	49 (22.50%)	0 (0.00%)	1 (12.50%)	
Code trauma	78 (11.50%)	102 (46.80%)	3 (100.00%)	5 (62.50%)	
Trauma type^b^					.004**^d^
Blunt	664 (97.30%)	205 (94.00%)	2 (66.70%)	6 (75.00%)	
Penetrating	17 (2.50%)	12 (5.50%)	1 (33.30%)	2 (25.00%)	
Burn	1 (0.10%)	1 (0.50%)	0 (0.00%)	0 (0.00%)	
Injury region: face, head, neck^b^					.864^d^
Yes	322 (47.20%)	121 (55.50%)	2 (66.70%)	4 (50.00%)	
No	360 (52.80%)	97 (44.50%)	1 (33.30%)	4 (50.00%)	
Transfer in^b^					.050*^d^
Yes	108 (15.80%)	15 (6.90%)	1 (33.30%)	1 (12.50%)	
No	573 (84.20%)	203 (93.10%)	2 (66.70%)	7 (87.50%)	
Time from injury to ED^b^					.057^d^
Less than 30 minutes	66 (9.70%)	26 (11.90%)	1 (33.30%)	2 (25.00%)	
31- 60 minutes	300 (44.00%)	108 (49.50%)	1 (33.30%)	2 (25.00%)	
61-90 minutes	53 (7.80%)	12 (5.50%)	1 (33.30%)	1 (12.50%)	
91 minutes-12 hours	127 (18.60%)	26 (11.90%)	0 (0.00%)	1 (12.50%)	
Greater than (>) 12 hours	136 (19.90%)	37 (17.00%)	0 (0.00%)	2 (25.00%)	
Pre-existing comorbidities^b^					.065^d^
Yes	153 (22.40%)	56 (25.70%)	2 (66.70%)	4 (50.00%)	
No	529 (77.60%)	162 (74.30%)	1 (33.30%)	4 (50.00%)	
ED BMI^a^	26.30 (SD=5.83)	27.84 (SD=8.67)	27.04 (SD=3.14)	27.72 (SD=5.51)	.042*^c^
ED BMI over (>) 40^b^					.272^d^
Yes	9 (1.40%)	7 (3.60%)	0 (0.00%)	0 (0.00%)	
No	615 (98.60%)	186 (96.40%)	3 (100.00%)	8 (100.00%)	
ED ISS^a^	11.52 (SD=6.55)	17.24 (SD=12.42)	28.33 (SD=13.65)	24.75 (SD=13.86)	<.001***^c^
ED ISS above 15^b^					<.001***^d^
Yes	173 (25.50%)	111 (51.40%)	3 (100.00%)	5 (62.50%)	
No	505 (74.50%)	105 (48.60%)	0 (0.00%)	3 (37.50%)	
ED serum lactate^a^	3.43 (SD=3.40)	4.88 (SD=4.17)	10.89 (SD=7.70)	2.50 (SD=1.11)	<.001***^c^
ED platelet count^a^					.288^c^
ED arrival GCS^a^	14.48 (SD=1.52)	10.83 (SD=4.64)	8.33 (SD=6.11)	6.30 (SD=2.38)	<.001***^ c^
ED SBP^a^	137.50 (SD=26.21)	135.35 (SD=32.75)	66.67 (SD=58.60)	113.58 (SD=36.20)	<.001***^c^
ED DBP^a^	78.33 (SD=19.51)	82.99 (SD=25.46)	42.67 (SD=32.17)	76.14 (SD=24.63)	<.001***^c^
ED HR^a^	87.82 (SD=21.32)	96.83 (SD=26.49)	86.67 (SD=75.72)	93.29 (SD=33.52)	<.001***^c^
ED temperature (fahrenheit)^a^	97.92 (SD=0.97)	97.77 (SD=1.05)	97.67 (SD=1.16)	98.20 (SD=0.84)	.219^c^
ED HCT^a^	38.64 (SD=6.72)	40.24 (SD=6.59)	29.75 (SD=6.01)	38.22 (SD=5.70)	.020*^c^
ED INR^a^	1.27 (SD=1.30)	1.22 (SD=0.94)	1.10 (SD=0.81)	1.77 (SD=1.16)	.860^c^
ED BAL^a^	.105 (SD=.129)	.112 (SD=.142)	.160 (SD=.156)	.130 (SD=.183)	.801^c^
ED O_2_^a^	96.87 (SD=3.45)	95.95 (SD=5.49)	68.67 (SD=53.41)	93.43 (SD=8.00)	<.001***^c^
ED supplemental oxygen administered (O_2)_^b^					<.001***^d^
Yes	64 (9.40%)	134 (61.50%)	3 (100.00%)	3 (37.50%)	
No	618 (90.60%)	78 (35.80%)	0 (0.00%)	5 (62.50%)	
ED probability of survival^a^	.961 (SD=.043)	.841 (SD=.236)	.634 (SD=.537)	.622 (SD=.352)	<.001***^c^
Units of PRBC received in less than 24 hr^a^	0.35 (SD=1.09)	1.44 (SD=3.48)	14.67 (SD=22.03)	1.12 (SD=2.23)	<.001***^c^
Units of plasma received in less than 24 hr^a^	0.08 (SD=0.39)	0.62 (SD=2.15)	9.33 (SD=14.47)	1.50 (SD=2.33)	<.001***^c^
Units of platelets received in less than 24 hr^a^	0.20 (SD=0.53)	0.50 (SD=1.21)	2.00 (SD=3.46)	0.62 (SD=0.74)	<.001***^c^
Units of cryoprecipitate received in less than 24 hr^a^	0.04 (SD=0.09)	0.23 (SD=0.99)	2.00 (SD=3.51)	0 (SD=0.00)	<.001***^c^
Administration of VIIA V in less than 24 hr^a^	0.00 (0.00)	0.00 (0.00)	0.00 (0.00)	0.00 (0.00)	n/a^e^
Cellsaver in less than 24 hr^a^	0.00 (0.00)	0.00 (0.00)	0.00 (0.00)	0.00 (0.00)	n/a^e^
MTP^b^					<.001***^d^
Yes	4 (0.60%)	15 (6.90%)	2 (66.70%)	0 (0.00%)	
No	674 (99.40%)	202 (93.10%)	1 (33.30%)	8 (100.00%)	

**Table 3:** The participants' demographic and clinical characteristics as per airway management; ventilator days ≥ four days Early tracheostomy = tracheostomy less than 10 days after endotracheal intubation; late tracheostomy = tracheostomy 10 days and greater after endotracheal intubation; ^a^Mean, SD; ^b^Frequency, percentage; ^c^One-way analysis of variance estimates (ANOVA) (f statistic) were conducted for continuous variable; ^d^Crosstabulation chi-square estimates were conducted for categorical data (except when expected count < 5 in a cell Fisher’s exact statistic was estimated); ^e^Inferential statistic not calculated; The chosen critical alpha level for all significance tests was 0.05 or less; *p values significant at 0.05 or <; **p values significant at 0.01 or <; ***p values significant at 0.001 or < SD: standard deviation; ED: emergency department; BMI: body mass index; ISS: Injury Severity Score; GCS: Glasgow Coma Score; SBP: systolic blood pressure; DBP: diastolic blood pressure; HR: heart rate; HCT: hematocrit; INR: international normalized ratio; BAL: blood alcohol level; O2: oxygen saturation; PRBC: packed red blood cells; MTP: massive transfusion protocol

Characteristics	Endotracheal intubation only (n=109)	Early tracheostomy (n=2)	Late tracheostomy (n=8)	p-value^c,d,e^
Sex^b^				.850^d^
Male	94 (86.20%)	2 (100.00%)	7 (87.50%)	
Female	15 (13.80%)	0 (0.00%)	1 (12.50%)	
Age^a^	54.32 (SD=22.61)	39.00 (SD=2.83)	48.67 (SD=15.92)	.551^c^
Race^b^				.938^d^
Asian	4 (5.80%)	0 (0.00%)	0 (0.00%)	
Black	10 (14.50%)	0 (0.00%)	1 (12.5%)	
White	40 (58.00%)	1 (50.00%)	5 (62.5%)	
Other	15 (21.70%)	1 (50.00%)	2 (25%)	
Ethnicity^b^				.853^d^
Hispanic	52 (47.70%)	1 (50.00%)	3 (37.50%)	
Non-Hispanic	57 (52.30%)	1 (50.00%)	5 (62.50%)	
Extrication^b^				.109^d^
Yes	6 (5.70%)	0 (0.00%)	2 (25.00%)	
No	99 (94.30%)	2 (100.00%)	6 (75.00%)	
Prehospital arrest^b^				.787^d^
Yes	5 (4.60%)	0 (0.00%)	0 (0.00%)	
No	104 (95.40%)	2 (100.00%)	8 (100.00%)	
Trauma team preactivation^b^				.122^d^
Yes	38 (34.90%)	2 (100.00%)	4 (50.00%)	
No	71 (65.10%)	0 (0.00%)	4 (50.00%)	
Trauma activation level^b^				.668^d^
Not activated	7 (6.40%)	0 (0.00%)	0 (0.00%)	
Trauma consult	28 (27.50%)	0 (0.00%)	2 (25.00%)	
Trauma alert	27 (24.80%)	0 (0.00%)	1 (12.50%)	
Code trauma	47 (43.10%)	2 (100.00%)	5 (62.50%)	
Trauma type^b^				.053^d^
Blunt	102 (93.60%)	2 (66.70%)	6 (75.00%)	
Penetrating	6 (5.50%)	1 (33.30%)	2 (25.00%)	
Burn	1 (0.90%)	0 (0.00%)	0 (0.00%)	
Injury region: face, head, neck^b^				.916^d^
Yes	62 (56.90%)	1 (50.00%)	4 (50.00%)	
No	47 (43.10%)	1 (50.00%)	4 (50.00%)	
Transfer in^b^				.007**^d^
Yes	4 (3.70%)	1 (50.00%)	1 (12.50%)	
No	105 (96.30%)	1 (50.00%)	7 (87.50%)	
Time from injury to ED^b^				.332^d^
Less than 30 minutes	17 (15.60%)	0 (0.00%)	2 (25.00%)	
31- 60 minutes	57 (52.30%)	1 (50.00%)	2 (25.00%)	
61-90 minutes	5 (4.60%)	1 (50.00%)	1 (12.50%)	
91 minutes-12 hours	16 (14.70%)	0 (0.00%)	1 (12.50%)	
Greater than (>) 12 hours	12 (11.00%)	0 (0.00%)	2 (25.00%)	
Pre-existing comorbidities^b^				.328^d^
Yes	79 (72.50%)	1 (50.00%)	4 (50.00%)	
No	30 (27.50%)	1 (50.00%)	4 (50.00%)	
ED BMI^a^	28.21 (SD=6.67)	28.46 (SD=2.75)	27.72 (SD=5.51)	.991^c^
ED BMI over (>) 40^b^				.825^d^
Yes	4 (3.60%)	0 (0.00%)	0 (0.00%)	
No	93 (96.40%)	2 (100.00%)	8 (100.00%)	
ED ISS^a^	21.47 (SD=8.50)	21.01 (SD=7.072)	24.75 (SD=3.86)	.730^c^
ED ISS above 15^b^				.524^d^
Yes	66 (60.60%)	3 (100.00%)	5 (62.50%)	
No	43 (39.40%)	0 (0.00%)	3 (37.50%)	
ED serum lactate^a^	5.73 (SD=5.13)	10.89 (SD=7.70)	2.50 (SD=1.11)	.384^c^
ED platelet count^a^	152.90 (SD=68.32)	224.36 (SD=18.44)	280.50 (SD=34.67)	.206^c^
ED arrival GCS^a^	11.07 (SD=4.69)	11.00 (SD=5.66)	9.71 (SD=6.29)	.768^c^
ED SBP^a^	139.56 (SD=37.46)	100 (SD=14.14)	113.58 (SD=36.20)	.144^c^
ED DBP^a^	91.56 (SD=28.01)	64.00 (SD=5.66)	76.14 (SD=24.63)	.509^c^
ED HR^a^	102.78 (SD=32.18)	86.67 (SD=75.72)	93.29 (SD=33.52)	.219^c^
ED temperature (Fahrenheit)^a^	97.68 (SD=0.67)	98.02 (SD=1.41)	98.20 (SD=0.84)	.555^c^
ED HCT^a^	40.59 (SD=6.59)	29.75 (SD=6.01)	38.22 (SD=5.70)	.118^c^
ED INR^a^	1.12 (SD=0.145)	1.10 (SD=0.81)	1.77 (SD=1.16)	.806^c^
ED BAL^a^	.094 (SD=.093)	.160 (SD=.156)	.130 (SD=.183)	.530^c^
ED O_2_^a^	95.74 (SD=5.32)	99.50 (SD=7.07)	93.43 (SD=8.00)	.564^c^
ED supplemental oxygen administered (O_2)_^b^				.120^d^
Yes	34 (32.40%)	3 (100.00%)	3 (37.50%)	
No	71 (67.60%)	0 (0.00%)	5 (62.50%)	
ED probability of survival^a^	.794 (SD=.274)	.943 (SD=.060)	.622 (SD=.352)	.182^c^
Units of PRBC received in less than 24 hr^a^	2.07 (SD=4.34)	20.00 (28.28)	1.12 (SD=2.23)	<.001***^c^
Units of plasma received in less than 24 hr^a^	0.77 (SD=2.08)	13.00 (SD=18.38)	1.50 (SD=2.33)	<.001***^c^
Units of platelets received in less than 24 hr^a^	0.72 (SD=1.13)	3.00 (SD=4.24)	0.62 (SD=0.74)	.131^c^
Units of cryoprecipitate received in less than 24 hr^a^	0.32 (SD=0.99)	3.00 (SD=4.25)	0 (SD=0.00)	.021*^c^
Administration of VIIA V in less than 24 hr^a^	0.00 (0.00)	0.00 (0.00)	0.00 (0.00)	n/a^e^
Cellsaver in less than 24 hr^a^	0.00 (0.00)	0.00 (0.00)	0.00 (0.00)	n/a^e^
MTP^b^				.094^d^
Yes	10 (9.30%)	1 (50.00%)	0 (0.00%)	
No	98 (90.70%)	1 (50.00%)	8 (100.00%)	

However, the early tracheostomy patients did have a significant difference in the units of packed red blood cells, plasma, and cryoprecipitate in the first 24 hours of admission as compared to endotracheal intubation and late tracheostomy patients (p < .001, p < .001, and p = .094, respectively). When comparing the advanced airway groups on the primary outcome of ventilator days (Table 4), there was a significant difference between endotracheal intubation, 8.19 (SD=12.48); early tracheostomy, 3.67 (SD=2.52); and late tracheostomy, 25.75 (SD=14.24); P<.001). Additionally, there was a significantly higher rate of readmission within 30 days for early tracheostomy patients as compared to intubation and late tracheostomy groups (p = .003). However, on the secondary outcomes of pneumonia and mortality, there were no significant differences noted (p = .856 and p = .167, respectively). For the exploratory analysis of associations between known clinical risk factors (ISS, ED systolic blood pressure (SBP), and pre-existing comorbidities present) and SDoH (current smoker and functional dependence) and complications of pneumonia, readmission, and mortality, within trauma patients requiring advanced airway management, significant correlations were found (Table 5).

**Table 4:** Outcomes of interest between advanced airway management approaches Early tracheostomy = tracheostomy less than 10 days after endotracheal intubation; late tracheostomy = tracheostomy 10 days and greater after endotracheal intubation; ^a^Mean, SD. ^b^Frequency, percentage; ^c^One-way analysis of variance estimates (ANOVA) (f statistic) were conducted for continuous variable; ^d^Crosstabulation chi-square estimates were conducted for categorical data (except when expected count < 5 in a cell Fisher’s exact statistic was estimated); The chosen critical alpha level for all significance tests was 0.05 or less; *p values significant at 0.05 or <; **p values significant at 0.01 or <; ***p values significant at 0.001 or < LOS: length of stay

Outcome variables	Endotracheal intubation only (n=217)	Early tracheostomy (n=3)	Late tracheostomy (n=8)	p-value^c,d^
Ventilator days^a^	8.19 (12.48)	3.67 (2.52)	25.75 (14.24)	<.001***^c^
Pneumonia^b^	6 (2.80%)	0 (0.00%)	0 (0.00%)	.856^d^
Hospital LOS^a^	18.48 (22.85)	23.33 (24.79)	40.25 (15.09)	.028*^c^
Readmission^b^	5 (2.30%)	1 (33.30%)	0 (0.00%)	.003**^ d^
Mortality^b^	68 (31.20%)	1 (33.30%)	0 (0.00%)	.167^d^

**Table 5:** Correlations between complications and clinical and social determinants of health (SDoH) variables within advanced airway management participants *p values significant at 0.05 or <; ***p values significant at 0.001 or < ISS: Injury Severity Score; ED: emergency department; SBP: systolic blood pressure (arrival)

	ISS	ED SBP	Pre-existing comorbidities	Smoking	Functional dependence
Pneumonia	.122*	-.147*	.218*	.211*	.301*
Readmission	-.166*	-.185*	.158*	.171*	.208*
Mortality	.251***	-.270***	.182*	n/a	n/a

## Discussion

As expected, the results of univariate analysis revealed significant differences in clinical variables between patients requiring advanced airway management and those who did not. The findings of the advanced airway management patients having a higher percentage of trauma alert pre-activation, lower ED arrival GCS scores, and higher ED ISS values as compared to non-invasive airway management groups reflect the clinical severity of these patients and the associated need for airway management. Not surprisingly, after the advanced airway management groups were limited to those requiring mechanical ventilation for ≥ four days, comparable groups for inferential analyses on primary and secondary outcomes of interest were obtained. Similar to prior studies, our findings of a shorter hospital LOS in the early tracheostomy group as compared to the late tracheostomy group (23.33, SD=24.78; 40.25, SD=15.09, respectively) [3,4]. However, the small sample size and its associated wide confidence intervals around the point estimates limited power and increased the probability of a Type II error. The small early tracheostomy sample size limited the exploration of the differences in outcomes related to the mechanism of injury described in prior studies [7,8]. Specifically in our study, two patients had the tracheostomy performed within 24 hours of the initial ED encounter, while the other patient had a tracheostomy procedure five days after ED arrival. The two patients who underwent tracheostomy within 24 hours of initial ED arrival had different mechanisms of injury: one was a penetrating mechanism of injury and the other a blunt trauma. The penetrating injury was a result of a firearm discharge with a laceration of the femoral artery and bowel perforation as a result. The other was an unspecified blunt trauma to the head of other means (non-fall and non-motor vehicle collision) with a resulting large subdural hematoma. The patient who had the early tracheostomy on day five of mechanical ventilation experienced a blunt trauma mechanism of injury. This patient had a velocity-related fall from a significant height with a compression blunt trauma injury that resulted in a large bowel laceration with perforation. However, as found in prior retrospective studies, patients in our sample undergoing early tracheostomy were younger, each also having an injury severity score > 15, and markedly fewer comorbidities (one patient of the three was a current smoker), as compared to patients with late tracheostomy and endotracheal intubation airway management groups [2,9,10]. The need for a significant increase in the average units of packed red blood cells, plasma, and cryoprecipitate in the first 24 hours of admission for the early tracheostomy group, as compared to endotracheal intubation and late tracheostomy patients, was most likely a result of the hemorrhage experienced by each patient.

Similar to prior retrospective findings, there was a significantly longer amount of ventilator days (the primary outcome) in the late tracheostomy group as compared to early tracheostomy and intubation-only groups. As the average number of days to tracheostomy in the late tracheostomy group was 15.21 days, the number of ventilator days was Additionally, there was a significantly higher rate of readmission within 30 days for early tracheostomy patients as compared to intubation and late tracheostomy groups (p = .003). However, on the secondary outcomes of pneumonia and mortality, there were no significant differences noted (p = .856 and p = .167, respectively). For the exploratory analysis of associations between known clinical risk factors (ISS, ED SBP, and pre-existing comorbidities present) and SDoH (current smoker and functional dependence) and complications of pneumonia, readmission, and mortality, within trauma patients requiring advanced airway management, significant correlations were found (Table 5). However, further exploration of other SDoH identified to impact complications in trauma patients still needs exploration within a larger sample [20-22].

Limitations

Although the clinical and SDoH factors associated with complications within approaches to advanced airway management identified in this study show promise as possible variables correlated with complications in a clinical setting, there are limitations that merit discussion at this time. First, the study’s small sample size of patients experiencing a tracheostomy limited inference both within the study and the generalizability of findings. Further, the retrospective design of the study is limiting regarding the comparison of the groups and potentially unidentified confounding covariates. The exclusion of unidentified confounding covariates is a limitation of all inferential studies where an exhaustive list of variables that may impact the relationship estimates between observed variables has not been included in the analysis due to lack of data or insufficient power due to limited sample size. Although our study identified interesting variables associated with complications related to advanced airway management approaches, a large-scale multicenter study is needed to determine the validity and generalizability of these findings across different adult critical care populations. Additionally, exploration of other clinical and SDoH variables identified in prior studies to be associated with complications in advanced airway management will allow for associative comparison across the different critical care populations and more conclusive findings to inform future predictive risk models and interventions to prevent these complications. For example, social isolation, lack of access to transportation, and food insecurity have been associated with complications such as readmission. However, our data, due to the retrospective study design, did not capture these variables or reliable proxy variables of these SDoH to include in the analysis. Furthermore, simply identifying which patients had risk factors predictive of ICU admission does not imply that any interventions (such as early recognition, resource allocation, or targeted treatment approaches) will have any effect on important outcomes such as mortality, ventilator days, pneumonia, or LOS. The answers to those questions will require further studies after exploratory and predictive models have been found to be valid and reproducible.

## Conclusions

The findings in this study are important because they identify clinical and SDoH associated with complications after advanced airway management approaches within adult trauma patients. Additional research is needed to examine these associations within a large-scale multicenter sample that includes diverse adult critical care patients and other hypothesized clinical and SDoH associated with these complications.
